# Transcription Factors Involved in the Development and Prognosis of Cardiac Remodeling

**DOI:** 10.3389/fphar.2022.828549

**Published:** 2022-02-02

**Authors:** Jia-Hui Hong, Hai-Gang Zhang

**Affiliations:** Department of Pharmacology, College of Pharmacy, Army Medical University (Third Military Medical University), Chongqing, China

**Keywords:** transcription factors, cardiac hypertrophy, cardiac remodeling, cardiac fibrosis, fetal gene, autophagy, apoptosis

## Abstract

To compensate increasing workload, heart must work harder with structural changes, indicated by increasing size and changing shape, causing cardiac remodeling. However, pathological and unlimited compensated cardiac remodeling will ultimately lead to decompensation and heart failure. In the past decade, numerous studies have explored many signaling pathways involved in cardiac remodeling, but the complete mechanism of cardiac remodeling is still unrecognized, which hinders effective treatment and drug development. As gene transcriptional regulators, transcription factors control multiple cellular activities and play a critical role in cardiac remodeling. This review summarizes the regulation of fetal gene reprogramming, energy metabolism, apoptosis, autophagy in cardiomyocytes and myofibroblast activation of cardiac fibroblasts by transcription factors, with an emphasis on their potential roles in the development and prognosis of cardiac remodeling.

## 1 Dual Roles of Cardiac Remodeling

The basic function of the heart is pumping blood to peripheral organs to maintain their different physiological functions. The heart must work harder in the presence of an increasing workload than under normal conditions. In response to harder work, some structural changes happen in heart, indicated by size increasing and shape changing. This process is called cardiac remodeling. During cardiac remodeling, cardiomyocytes are centric cells, while other cells, like fibroblasts, also participate in this process. Both types of cardiac remodeling, i.e., physiological and pathological, are adaptive changes that maintain cardiac output under cardiac stress, but they differ in terms of the cellular mechanism and consequences (Wu et al., 2017). Physiological remodeling is characterized by a preserved or increased contractile ability induced by reversible cardiomyocyte growth in both width and length, and slight increases in heart mass without fibrosis and cell death. This type of harmless and adaptive cardiac hypertrophy often happens in growth, practice, and pregnancy (Schuttler et al., 2019). Conversely, pathological remodeling is characterized by a dilated ventricle with a thinner wall induced by increasing the cardiomyocyte length. Consequently, it progresses to systolic dysfunction, cardiac fibrosis, and heart failure. Multiple risk factors for pathological remodeling have been identified, such as hypertension, aortic stenosis, myocardial infarction, genetic cardiomyopathy and several systemic diseases (Wu et al., 2017).

As mentioned above, cardiomyocyte play a critical role in cardiac remodeling. In pathological condition, increasing cardiomyocytes cell sizes lead to heart size increase, which is called cardiac hypertrophy. Pathological cardiac hypertrophy often indicates a poor prognosis. Initially, the hypertrophic response increases myocardial contractility, reduces wall stress and maintains cardiac output through compensatory mechanisms. However, the continued presence and evolving hypertrophy eventually lead to decompensation of heart function, resulting in heart failure, malignant arrhythmia, and even sudden death (Oldfield et al., 2020; Liu et al., 2021). According to an investigation by the Sarcomeric Human Cardiomyopathy Registry with 24,000 person-years of follow-up, patients with cardiac hypertrophy had an approximately 3-fold higher mortality risk compared with similarly aged individuals in the United States general population (Virani et al., 2020). In addition, patients often suffer from a substantial economic burden and have a low quality of life. However, no effective cure is available to reverse pathological cardiac hypertrophy.

Besides cardiomyocytes, cardiac fibroblasts also participate in cardiac remodeling process with critical function. In physiological condition, cardiac fibroblast is an essential cell type in heart, which is mainly responsible for homeostasis of extracellular matrix protein (ECM). Pathologically, activated cardiac fibroblasts, also known as myofibroblasts, secret excessive ECM. Due to the limited repairment capacity of heart, excessive ECM accelerates the progression to heart failure (Kong et al., 2014). Like pathological cardiac hypertrophy, cardiac fibrosis also is an irreversible process with limited clinical treatment assays.

## 2 Function of Transcription Factors in Cardiac Remodeling

**FIGURE 1:**
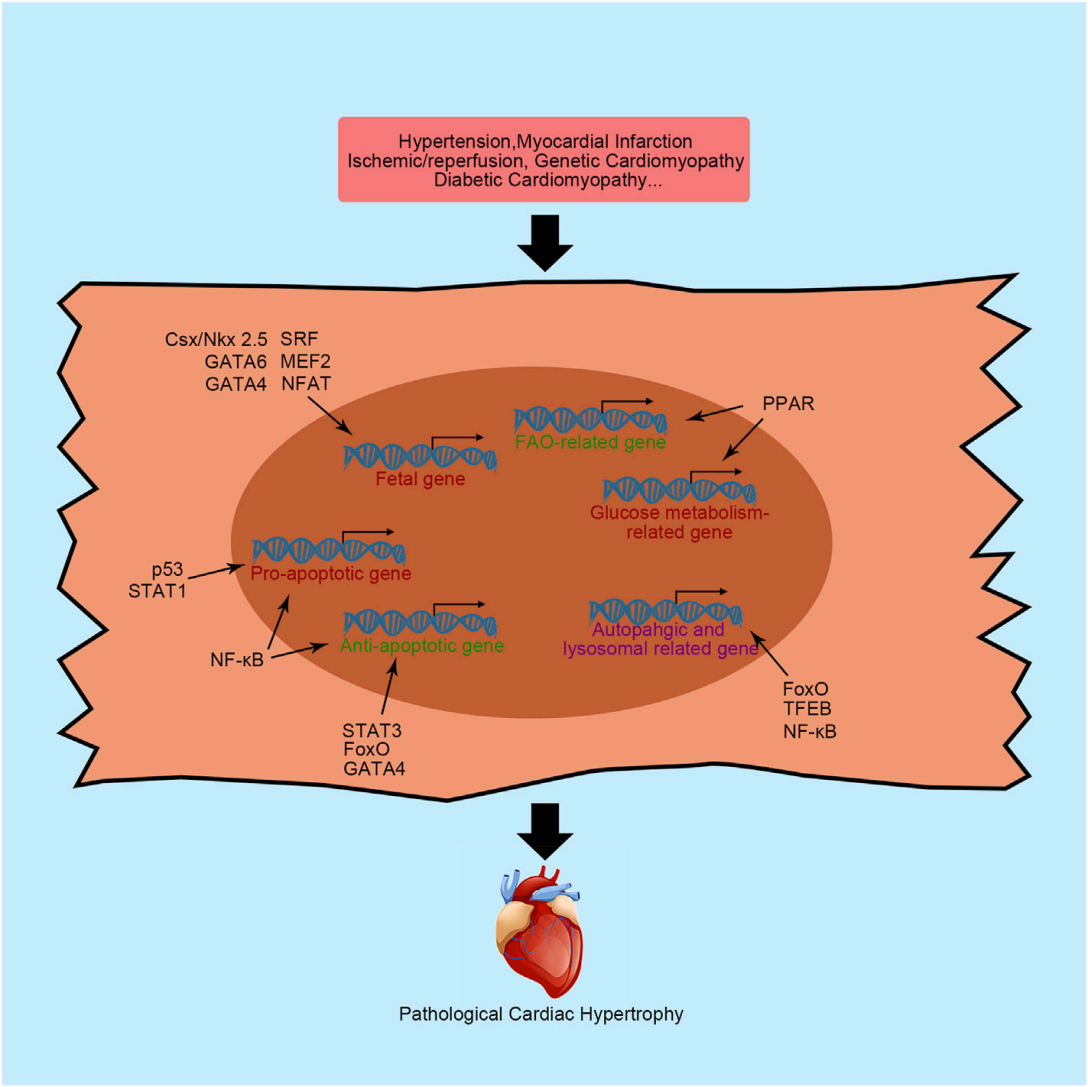
Primary transcription factors and their functions in cardiac hypertrophy. NFAT, nuclear factor of activated T-cells; MEF2, myocyte enhancer factor 2; Csx/NKX2-5, NK-2 transcription factor related, locus 5; SRF, serum response factor; β-MHC, cardiac muscle β-isoform; ANP, natriuretic peptide A; BNP, natriuretic peptide B; PPAR, peroxisome proliferator activator receptor; FAO, fatty acid oxidation; NF-κB, nuclear factor κB; STAT, signal transducers and activators of transcription; FoxO, forkhead domain transcription factor O; TFEB, transcription factor EB; SMAD, small mothers against decapentaplegic; MRTF, myocardin-related transcription factor.

**FIGURE 2:**
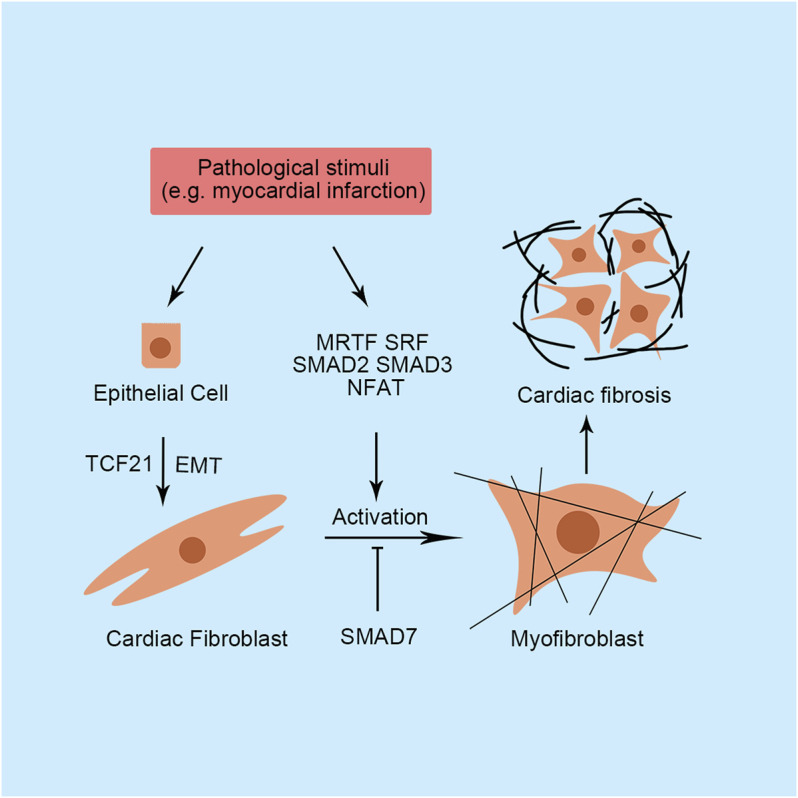
Primary transcription factors and their functions in cardiac fibrosis. SMAD, small mothers against decapentaplegic; SRF, serum response factor; MRTF, myocardin-related transcription factor; NFAT, nuclear factor of activated T-cells; TCF21, transcription factor 21; EMT, epithelial-to-mesenchymal transition.

Many studies have indicated that several critical signaling pathways are responsible for the remodeling process, such as the calcineurin/nuclear factor of activated T-cells (NFAT) pathway, Janus kinase/signal transducer and activator of transcription (STAT) pathway, and mitogen-activated protein kinase (MAPK) pathway (Shimizu and Minamino, 2016). Activation of these pathways requires extracellular hypertrophic stimulation, such as pressure overload and energy starvation, and results in changes in the transcriptional program and the alternative synthesis of nucleic acids and proteins. Therefore, transcription factors play a critical role in the regulation of cardiac hypertrophy because they directly bind to DNA sequences within promoters or enhancers to control transcription. A cardiac transcription factor is defined as a protein that regulates transcription in the heart to ensure that genes are expressed in the right cell at the right time in the right amount. Currently, numerous studies have established that cardiac transcription factors influence the cardiac remodeling process by regulating hypertrophy-related gene expression or fibroblasts activation-related gene expression (Figures 1, 2, and Table 1). In this review, we outline some important transcription factors regulating fetal genes, energy metabolism, apoptosis, and autophagy in cardiomyocytes and activation in cardiac fibroblasts, which may provide a new perspective for further exploration.

**TABLE 1:** Transcription factors and their primary target genes in the regulation of cardiac remodeling.

Function	Transcription factor	Target genes	References
Expression of fetal genes	NFAT	*Nppb*, *Adss1*, *Ppp3cb*, miR-25, miR-23a, and miR-199b	Molkentin et al. (1998), Luo et al. (2021)
	GATA	*Nppa*, *Nppb*, *Myh7*, *Myh6*, *Tnnc1*, *Tnni1*, *Ncx*, *Adora1*, *Chrm2*, *Mlc1* and *Mlc3*	Zeisberg et al. (2005)
	MEF2	*Nppa* and *Myh7*	Zuo et al. (2015)
	Csx/NKX2-5	*Nppa*, *Adss1*, *Calr*, *Gja5*, *Ncx* and *Mef2c*	Lints et al. (1993)
	SRF	*c-fos*, *Nppa*, *Nppb*, *Myh6*, *Myh7*, and *Ncx1*	Nelson et al. (2005)
Energy metabolism	PPARα	*Acox1*, *Cd36*, *Cpt1b*, *Acsl3*, *Cpt1a*, *Hmgcs2*, *Pcx*, and *Plin2*	Oka et al. (2015)
Apoptosis	NF-κB	*Bax*, *Fas*, *Faslg*, *Tp53*, and miR-30b	Dolcet et al. (2005), Wei et al. (2014)
	STAT1	*Fas* and *Faslg*	Eid et al. (2018)
	STAT3	*Bcl2l1* and *Sod*	Harhous et al. (2019)
	p53	*Bax* and lncRNA-Meg3	Gogna et al., 2013; Li X et al. (2019)
	FoxO	*Bnip3*, *Bax*, *Sod*, *Cited2* and ARC	Sengupta et al. (2011), Lu et al. (2013)
	GATA4	*Bcl2*	Tony et al. (2015)
Autophagy	TFEB	*Atg9*. *Vps11*, and *Lamp1*	Settembre and Ballabio (2011)
	NF-κB	*Becn1*	Zeng et al. (2013)
	FoxO	*Atg8*, *Atg12*, *Atg4b*, *Pik3c3*, *Gabarapl1* and *Becn1*	Ferdous et al. (2010)
Fibrosis	SMAD	*Col1a*, *Acta2* and *Tagln*	Lighthouse and Small (2016)
	MRTF	*Col1a2*	Small et al. (2010)
	NFAT	*Col3* and *Mrtfa*	Herum et al. (2013)
	SRF	*Acta2 and Col1*	Bretherton et al. (2020)

NFAT, nuclear factor of activated T-cells; MEF2, myocyte enhancer factor 2; Csx/NKX2-5, NK-2 transcription factor related, locus 5; SRF, serum response factor; PPARα, peroxisome proliferator activator receptor α; NF-κB, nuclear factor κB; STAT, signal transducer and activator of transcription; FoxO, forkhead domain transcription factor O; ARC, apoptosis repressor with caspase recruitment domain; TFEB, transcription factor EB; SMAD, small mothers against decapentaplegic; MRTF, myocardin-related transcription factor.

## 3 Profile and Mechanism of Transcription Factors in the Process of Cardiac Remodeling

### 3.1 Transcriptional Regulation of the Fetal Gene Program

Pathological cardiac hypertrophy often manifests as the reactivation of a “fetal gene program” characterized by the upregulated expression of fetal genes, including β-isoform of cardiac myosin heavy chain (β-MHC), atrial natriuretic peptide (ANP), and brain natriuretic peptide (BNP). Although the activation of these genes seems to be salutary at the early stage of cardiac hypertrophy, the aberrant expression of fetal genes results in abnormal function of cardiac contractility, calcium hemostasis, cell growth and finally heart dysfunction. Therefore, the amplified fetal genes expression helps doctors to assess cardiac function (Ingles et al., 2019). For instances, NT-proBNP has been identified as an accessory biomarker for assessing cardiac function. Interestingly, when cardiac hypertrophy is ameliorated, fetal gene expression will be significantly reduced. Here, we will introduce some transcription factors governing the expression of fetal genes.

#### 3.1.1 Nuclear Factor of Activated T-Cells

NFAT is an important protein involved in an intercellular signaling pathway that regulates the expression of fetal genes. The calcineurin-regulated NFAT family consists of five family members, NFAT1-5. All of them are expressed in the heart. After dephosphorylation by calcineurin, NFAT translocates into the nucleus, binds to the specific sequence and recruits transcriptional elements. Subsequently, fetal genes are re-expressed and activated. The function of NFAT in embryos is well understood. The lack of NFAT in fetal development induces a loss of contractile protein and a thin atrial wall in mice (Schubert et al., 2003). However, when this fetal gene program governor is reactivated in the adult mammalian heart, a hypertrophic response occurs concomitantly. In human, the increasing dephosphorylation of NFAT3 is related with dilated cardiomyopathy (DCM) (Diedrichs et al., 2004). Suppression of NFAT activity by nuclear localized protein 1 effectively attenuates isoprenaline-induced cardiac hypertrophy in mice, as indicated by reduced cardiac fibrosis, improved cardiac contractility function and preservation of left ventricular geometry, which suggested the harmful role of NFAT in the adult heart (Zhang et al., 2020). *Nppb* is one of the genes regulated by NFAT in cardiomyocytes. Using neonatal rats ventricular myocytes (NRVMs), Molkentin et al. (1998) found that the *Nppb* promoter was activated 100-fold more in the presence of NFAT with the coactivator GATA-4. Furthermore, overexpression of calcineurin leading to constitutive activation of NFAT dramatically increases the expression of BNP transcripts and induces serious cardiac hypertrophy and fibrosis in mice. In addition to *Nppb*, regulator of calcineurin 1.4 is validated as a direct target of NFAT in NRVMs (Luo et al., 2021). Recently, the role of NFAT in epigenetics was noted. NFAT controls many microRNAs transcription, such as miR-23a and miR-223 (Luo et al., 2021). These microRNAs controlled by NFAT are positively correlated with pathological cardiac hypertrophy (Chen et al., 2017).

#### 3.1.2 GATA Transcription Factors

GATA is another transcription factor superfamily that exerts critical regulatory functions in the heart. Six known members of the GATA family have been detected in vertebrates. GATA1/2/3 are preferentially expressed in hematopoietic cells, while GATA4/5/6 proteins are expressed in various tissues, including the liver, lung, gut, gonad and heart. Among the GATA superfamily members, GATA4 is the most extensively explored transcription factor in the heart (Whitcomb et al., 2020). Similar to NFAT, inactivation of GATA4 in mouse embryos leads to death by E8.5 due to abnormal heart development (Zeisberg et al., 2005). Combined with other transcription factors, GATA4 directly regulates the expression of numerous genes in the heart, including *Nppa*, *Nppb*, *Myh7*, *Myh6*, *Tnnc1*, *Tnni1*, *Ncx*, *Adora1*, *Chrm2*, *Mlc1*, and *Mlc3* (Zeisberg et al., 2005). Upregulated GATA4 expression has been found in human DCM heart (Diedrichs et al., 2004). In adult hearts, transgenic mice with cardiomyocytes-specific overexpression of GATA4 by 2.5-fold displayed cardiac hypertrophy (Liang et al., 2001). Expression of a dominant negative GATA4 by a GATA4 engrailed repressor fusion-encoding adenovirus ameliorates phenylephrine-induced cardiac hypertrophy and remodeling in NRVMs (Liang et al., 2001). The mechanism was further studied by mutating serine 105 to alanine in GATA4 (GATA4-S105A), which is a known GATA4 phosphorylation site. GATA4-S105A mice did not exhibit cardiac hypertrophy induced by 2 weeks of PE infusion with decreased expression of *Nppa*, *Nppb* and *Myh7*, signifying that extracellular signal-regulated kinase 1/2 and p38 MAPK dependent phosphorylation of GATA4 were required for fetal gene reactivity in hypertrophic hearts (van Berlo et al., 2011). In addition, acetylation was necessary for GATA4 to exert hypertrophic effects. Deacetylation of GATA4 by Sirtuin 7 significantly ameliorates transverse aortic constriction (TAC)-induced cardiac hypertrophy in mice (Yamamura et al., 2020).

Over 30 mutations in GATA6 gene are associated with congenital heart disease, indicating the pivotal regulating function of GATA6 in heart (Whitcomb et al., 2020). Both gain-of-function and loss-of-function studies have established a critical role for GATA6 in cardiomyocytes. Forced expression of GATA6 in mouse cardiomyocytes results in an increased cell surface area, while cardiomyocytes specific GATA6 deletion leads to decreased fetal gene expression and reduced cardiac hypertrophy caused by pressure overload stimulation in mice (van Berlo et al., 2010). The functions of GATA4 and GATA6 partially overlap. Neonatal GATA4 and GATA6 inactivation in mouse hearts causes more rapid and severe heart failure than single knockout (Liang et al., 2001). However, GATA4 and GATA6 do not completely compensate for each other. Hybridized mice with knockout of GATA4 or GATA6 and overexpression of the other protein displayed partially reversed cardiac hypertrophy without full heart function recovery (van Berlo et al., 2013).

#### 3.1.3 Myocyte Enhancer Factor 2

The myocyte enhancer factor 2 (MEF2) superfamily consists of four members, namely, MEF2A-D. During mouse embryonic development, MEF2B and MEF2C are first expressed in the cardiac mesoderm at E7.5, followed by MEF2A and MEF2D expression a day later (Desjardins and Naya, 2016). The heterozygous mutation of MEF2C in human increased susceptibility to DCM (Yuan et al., 2018). In addition, upregulation of MEF2 is related to cardiac hypertrophy in mice adult hearts. Several fetal genes have been identified as direct target genes of MEF2, including *Nppa* and *Myh7* (Zuo et al., 2015). Knockdown of MEF2C or MEF2D in mice is sufficient to blunt hypertrophic heart growth in response to pressure overload, while the overexpression of MEF2A, MEF2C and MEF2D causes ventricular dilation and cardiac dysfunction (Clapham et al., 2019). The robust activation of MEF2 in cardiac hypertrophy is associated with histone deacetylases (HDACs), which promote chromatin condensation and subsequent transcriptional repression (Zhang et al., 2002). Cardiac hypertrophic stimulation is sufficient to induce HDAC phosphorylation, which promotes the nuclear export of HDAC and transcriptional activity of MEF2. Mutation of the phosphorylation site of HDAC9 renders mouse cardiomyocytes intolerant to PE-induced cardiac hypertrophy and upregulates ANP and β-MHC expression. However, HDAC9-null mice display an increased sensitivity to hypertrophic stimuli, such as pressure overload, indicating that unphosphorylated HDAC continually suppresses MEF2 transcriptional activity (Zhang et al., 2002).

#### 3.1.4 NK-2 Transcription Factor Related, Locus 5

The homeodomain-containing transcription factor Csx/NKX2-5 belongs to the NK homeobox gene family, and functions as a transcriptional activator with an indispensable regulatory role in embryo heart development and adult heart function. The name of Csx/NKX2-5 consists of two parts. “Csx” is the acronym for cardiac-specific homeobox, indicating the critical biological function in myocardial cells, while NKX2-5 represents the fifth vertebrate gene identified in the NK-2 homeobox gene family. The critical role of Csx/NKX2-5 in developmental biology has been extensively established (Chen and Fishman, 1996). The direct downstream target genes of Csx/NKX2-5 have also been extensively explored, such as *Nppa*, *Adss1*, calreticulin, connexin40, *Ncx1*, and MEF2C (Lints et al., 1993). The regulatory mechanism of Csx/NKX2-5 often synergizes with other transcription factors, such as GATA4. Using COS-9 cell lines, Shiojima et al. (1999) found that expression of both Csx/NKX2-5 and GATA4 results in the much higher transcription of *Nppa* compared with Csx/NKX2-5 expression alone, indicating that Csx/NKX2-5 synergized with GATA4 to initiate *Nppa* transcription. In humans, mutations in Csx/NKX2-5 are strongly associated with congenital heart diseases (Wang et al., 2011). Taken together, this evidence supports a critical role for Csx/NKX2-5 in the fetal gene program and cardiac hypertrophy.

#### 3.1.5 Serum Response Factor

As previously mentioned, SRF also regulates fetal gene expression with the help of some cofactors, such as GATA4 and Csx/Nkx2-5. SRF has been discovered in regulating several gene expression including cell growth, differentiation, migration, etc., (Niu et al., 2005). Cardiac-specific knockout of SRF in embryos leads to cardiac insufficiency and embryonic lethality (Niu et al., 2005). In the postnatal heart, the presence of SRF is required for the expression of *c-fos*, *Nppa*, *Nppb*, *Myh6*, *Myh7*, and *Ncx1* (Nelson et al., 2005). However, the function of SRF in the regulation of cardiac hypertrophy is self-contradictory. Both gain and loss of SRF function induce cardiac hypertrophy (Liu et al., 2015). Using heart specific tamoxifen-induced Cre recombinase, cardiomyocytes specific disruption of SRF in mice causes impaired left ventricular function with decreased contractility. Besides, all mutant mice died from serious heart failure 10 weeks after treatment, indicating the harmful role of SRF in cardiomyocytes (Parlakian et al., 2005). However, transgenic mice with cardiomyocytes-specific overexpressing human SRF also leads to serious cardiac hypertrophy (Zhang et al., 2001). In addition, SRF expression levels are decreased in subjects with PE-induced cardiac hypertrophy but increased in aortic banding-induced cardiac hypertrophy (Nelson et al., 2005; Liu et al., 2015). Recently, Li et al. (2020) reported that phosphorylation of SRF significantly decreased in dilated cardiomyopathy patients. Besides, the activated caspase 3 cleaves SRF and produce a domain-negative transcription factor, suggesting that increasing SRF expression may benefit the DCM patients (Chang et al., 2003).

### 3.2 Regulation of Energy Metabolism and Peroxisome Proliferator Activator Receptors

The heart is a typical organ with a high energy consumption. Two primary sources of continuous and extensive energy production in cardiomyocytes have been shown to sustain the heartbeat: mitochondrial oxidative phosphorylation and glycolysis. The former accounts for approximately 95% of myocardial ATP consumption, while the latter accounts for approximately 5%. After exposure to different stresses, the heart utilizes diverse ranges of substrates to produce energy. In normal hearts, approximately 40–60% ATP is produced using fatty acids as fuel (Karwi et al., 2018). In addition, lactate, glucose, ketones, and amino acids are also common substrates for energy production in the heart. Generally, a dramatic alteration in energy metabolism occurs in hypertrophic and failing hearts (Karwi et al., 2018). This disordered energy metabolism is caused by several factors, including impaired mitochondrial oxidative metabolism, alterations in energy substrate preference by the heart, and a decrease in cardiac efficiency. A decrease in ATP production is a characteristic of the failing heart and is primarily caused by impaired mitochondrial oxidative capacity. Compared with a healthy heart, approximately 30% less ATP production is detected in the end-stage failing heart (Karwi et al., 2018). Furthermore, changes in the energy pattern are associated with alterations in the transcriptional program. Indeed, fatty acid oxidation (FAO)-related genes are downregulated in hypertrophic and failing hearts of hypertensive rats compared to controls (Karwi et al., 2018).

Alterations in cardiac energy production contribute to the severity of heart failure. The peroxisome proliferator activator receptor (PPAR) superfamily is an important and well-known protein superfamily affecting heart energy metabolism by regulating transcription. All three isoforms of PPAR, PPARα, PPARβ/δ, and PPARγ, are expressed in the heart. PPAR activation is characterized by the formation of transcriptional complexes with ligands, retinoic acid-x-receptors and coactivators. Subsequently, the active complex directly binds to the specific DNA binding element to recruit related enzymes, remodel chromatin, modify histones and initiate transcription (Ajith and Jayakumar, 2016).

#### 3.2.1 PPARα

The function of PPARα in the heart has been studied extensively using gain-of-function and loss-of-function methods. The rate of fatty acid metabolism decreases along with an increase in the glucose consumption rate in the hearts of PPARα^−/−^ mice (Oka et al., 2015). In addition, PPARα-deficient mice display reduced cardiac function and impaired compensation for an increased workload (Khuchua et al., 2018). This phenotype was partially caused by lipid metabolite accumulation and energy starvation. In PPARα-overexpressing mice, several PPAR target genes, such as *Acox1*, *Cd36*, *Cpt1a, Cpt1b*, *Acsl3*, *Hmgcs2*, *Pcx*, and *Plin2*, are upregulated (Oka et al., 2015). Fatty acid metabolism-related enzymes are upregulated along with decreased expression of glucose uptake- and oxidation-related proteins in mice with cardiomyocytes-restricted overexpression of PPARα (MHC-PPARα) (Finck et al., 2002). Using isolated working hearts and micro–positron emission tomography analyses, Finck et al. (2002) established that the myocardium predominantly uses fatty acids as energy resources instead of glucose uptake and metabolism when PPARα is overexpressed in cardiomyocytes. Taken together, these findings suggested that PPARα activation in cardiomyocytes increases fatty acid uptake, storage, and utilization by controlling the expression of some critical genes in the FAO pathway. However, researchers have been unable to conclude that downregulated PPARα is associated with cardiomyopathy. First, different levels of PPARα expression are observed in individuals with HF characterized by FAO deficiency, suggesting that downregulated PPARα is not a common feature of heart failure (Khuchua et al., 2018). Second, both cardiomyocytes-specific knockout and overexpression of PPARα induce cardiac hypertrophy in mouse hearts, which indicates the controversial role of PPARα in the development of cardiac hypertrophy (Finck et al., 2002; Khuchua et al., 2018). In addition, the activation of PPARα in different animal models leads to different consequences. Administration of WY14643, a specific PPARα ligand, in the early stage of severe myocardial damage induced by diabetes leads to a higher mortality rate than the vehicle group (Chen et al., 2010). However, PPARα activation in the early stage of TAC-induced mouse heart failure preserves myocardial function (Kaimoto et al., 2017). Last, the effect of PPARα on regulating Glut4, a glucose transporter promoting glucose uptake, remains controversial. In MHC-PPARα mice, the upregulated PPARα exerts an inhibitory effect on Glut4, while increased PPARα activation increases Glut4 expression synergistically with the PPARα coactivator fenofibrate after administration to mice, signifying the complicated role of PPARα in regulating cardiac glucose metabolism (Burkart et al., 2007; Yao et al., 2012). Taken together, all the studies conducted to date have revealed the complex regulatory mechanism of PPARα in the diseased heart.

#### 3.2.2 PPARβ/δ

Both PPARβ/δ and PPARγ play similar roles to PPARα in cardiomyocytes. All of these proteins govern FAO-related gene expression in cardiomyocytes, including fatty acid uptake, mitochondrial fatty acid β-oxidation, malonyl-CoA metabolism, and peroxisomal oxidation (Burkart et al., 2007). The specific deletion of PPARβ/δ in mouse cardiomyocytes leads to heart dysfunction and reduced fatty acid metabolism, signifying the regulatory effect of PPARβ/δ on FAO in the heart (Chen et al., 2013). Interestingly, PPARβ/δ activation increases FAO-related and glucose oxidation-related gene expression (Wang et al., 2010). As a result of these changes, cardiomyocytes-restricted PPARβ/δ overexpressing mice fed a high-fat diet do not have cardiac myopathy induced by lipid accumulation compared with MHC-PPARα diabetic mice (Burkart et al., 2007). Notably, the level of the endogenous antioxidant Cu/Zn superoxide dismutase is also increased after the constitutive activation of PPARβ/δ, indicating that PPARβ/δ activation protects the heart from oxidative stress (Barlaka et al., 2015). All this evidence suggests a protective role for PPARβ/δ in the heart. Indeed, the PPARβ/δ activation rescues in heart dysfunction in mice after ischemic/reperfusion (I/R) injury compared with wild-type mice, and PPARβ/δ activation protects neonatal rat cardiomyocytes from hypertrophy. Consistent with the *in vitro* findings, PPARβ/δ overexpression in the cardiomyocytes improves cardiac function and reduces fibrosis and energy metabolism in mice with pressure overload-induced cardiac hypertrophy (Wang et al., 2010). Therefore, the administration of PPARβ/δ ligands might be a potential therapeutic method for cardiomyopathy in the future.

#### 3.2.3 PPARγ

Many researchers have explored the function of PPARγ in cardiomyocytes. Compared with adipose-enriched tissue, PPARγ is expressed at a lower level in the heart. PPARγ agonists, such as thiazolidinediones, are widely used in the clinic as insulin sensitizers to treat diabetes and exhibit good efficacy in preventing cardiac vascular events. However, emerging evidence has shown that PPARγ agonists may not adequately protect the heart (Wright et al., 2014). In the human heart, upregulated PPARγ expression has been detected in the left ventricle and coronary arteries of patients with cardiomyopathy and coronary heart disease compared with normal people (Mehrabi et al., 2003). Paradoxically, Liu et al. (2016) revealed that quercetin protected the heart from I/R-induced damage *via* PPARγ activation in H9C2 cells, indicating the protective role of PPARγ in the heart. The conflicting results may be attributed to the off-target effects of PPARγ agonists. Cardiomyocytes-specific knockout of PPARγ leads to mild cardiac hypertrophy with preserved heart function (Duan et al., 2005). After treatment with rosiglitazone, a PPARγ agonist, cardiomyocytes-specific PPARγ-null mice display serious cardiac hypertrophy and cardiac dysfunction, indicating the indirect hypertrophic effect of PPARγ agonists (Duan et al., 2005). Using cardiac specific PPARγ overexpressing mice, Son et al. (2007) found that cardiomyocytes-specific overexpression of PPARγ caused all mice to die at 5 months of age from severe cardiac hypertrophy due to high fatty acid uptake and metabolism. Additionally, the expression of glucose uptake-related genes did not decrease. Interestingly, when cardiomyocytes-specific PPARγ-overexpressing mice were crossed with PPARα-deficient mice, cardiac hypertrophy was significantly ameliorated, along with upregulated FAO and decreased apoptosis, reactive oxygen species (ROS) levels, and endoplasmic reticulum stress (Son et al., 2010). These complex and incompatible findings regarding the function of PPARγ suggest that suitable expression of PPARγ benefits the heart. Researchers have not clearly determined whether excessively high or low PPARγ expression in cardiomyocytes induces cardiac hypertrophy and dysfunction.

### 3.3 Regulation of Apoptosis

Apoptosis refers to a process of programmed cell death. Physiologically, apoptosis plays a critical role in maintaining homeostasis by destroying unnecessary cells. However, excess apoptosis will result in pathological conditions and finally lead to organ failure. Researchers concluded that reduced cardiomyocytes apoptosis protects the heart from cardiac dysfunction and remodeling during pathological conditions, such as myocardial infarction (MI) and I/R injury (Teringova and Tousek, 2017). The expression of several apoptosis-related genes is upregulated in the cardiomyocytes under pathological conditions, while the expression of antiapoptotic genes is suppressed. These processes require the involvement of transcription factors. Here, we will introduce some critical transcription factors involved in regulating cardiomyocytes apoptosis.

#### 3.3.1 Nuclear Factor-κB

Nuclear factor-κB (NF-κB) is one of the core transcription factors in the apoptosis pathway and was first discovered to regulate immunoglobulin κB expression in B lymphocytes. The mammalian NF-κB superfamily contains five members, RelA (p65), RelB, c-Rel, p50 and p52, which are characterized by conserved Rel homology domain at the N-terminus. Further exploration suggested that NF-κB participates in various of cellular signaling pathways in multiple cell types (Zhang et al., 2017). Among all of these signaling pathways, inhibitor of nuclear factor κB (IκB) is a key regulator that directly controls NF-κB activation. Classically, NF-κB activation depends on the IκB kinase (IKK)-mediated phosphorylation of IκB. Subsequently, NF-κB translocates into the nucleus and binds to specific DNA sequences. NF-κB exerts a critical effect on regulating cardiomyocyte apoptosis, especially in response to oxidative stress following myocardial infarction (Imam et al., 2018). In human, Czibik et al. (2008) reported that NF-κB nuclear translocation was reduced at reperfusion in stable angina patients after ischemic precondition. However, researchers have not conclusively determined whether it exerts cardioprotective effects or deleterious effects. Indeed, NF-κB activation induces the expression of both proapoptotic proteins (e.g., Bax, Fas, FasL and p53) and antiapoptotic proteins (Dolcet et al., 2005). The cardiomyocytes-specific deletion of NF-κB essential modulator/IKKγ in mice causes spontaneous ventricular dilation with increased oxidative stress and apoptosis levels (Kratsios et al., 2010). Furthermore, the direct deletion of p50 in mice exacerbates cardiac ventricular dilation, inflammation and fibrosis (Timmers et al., 2009). However, in contrast, other studies reported that p50 deficiency in mice significantly improves survival and cardiac hypertrophy following infarction without decreasing inflammatory cytokine expression (Frantz et al., 2006). Using an IκBα mutation to specifically mask the nuclear localization sequence of p65, Hamid et al. found that reduced activity of p65 in mouse cardiomyocytes alleviates pathological cardiac hypertrophy by increasing stress-induced apoptosis (Hamid et al., 2011). In addition, they recognized that p65 is mainly responsible for NF-κB activity to initiate cardiac remodeling-related gene transcription (Hamid et al., 2011). Combined with the p50 deficiency-induced upregulation of p65 in fibroblasts from embryonic mice stimulated with lipopolysaccharide, controversial experimental results for p50 showed compensatory p65 activation (Timmers et al., 2009; Hamid et al., 2011). Currently, accumulating evidence has proven that NF-κB downregulation benefits the heart by reducing inflammation in both H9C2 cells, NRVMs and human (Mangali et al., 2019; Luo et al., 2020; Fan et al., 2021). Notably, NF-κB plays a critical role in epigenetics. By positively controlling miR-30b, NF-κB indirectly inhibits the expression of the antiapoptotic protein Bcl-2, resulting in increased NRVMs death upon angiotensin Ⅱ stimulation (Wei et al., 2014).

#### 3.3.2 Signal Transducer and Activator of Transcription

Another important transcription factor family governing apoptosis is STAT. Seven members of the STAT family have been identified in mammals, i.e., STAT1, STAT2, STAT3, STAT4, STAT5a, STAT5b and STAT6. Although they share similar structures, their biological functions are different. Among these seven members, the main STATs functioning in the heart are STAT1 and STAT3. Therefore, we will discuss these two transcription factors in more detail. Many early studies established that STAT1 and STAT3 play critical roles in controlling cell fate by regulating the expression of apoptosis-related genes. In human, the phosphorylation of both STAT1 and STAT3 are significantly increased in DCM (Ng et al., 2003). However, animal studies revealed that STAT1 and STAT3 exert completely opposite effects on controlling cardiomyocyte apoptosis, similar to their effects on cancer cells (Knight et al., 2012). The phosphorylation of STAT1, indicating activated STAT1, is increased in cardiomyocytes following I/R damage and upregulates proapoptotic proteins such as Fas and FasL (Eid et al., 2018). Moreover, STAT1 interacts with a well-known proapoptotic transcription factor, p53, to increase the transcriptional activity of p53 in apoptosis (Carroll et al., 2013). In contrast, STAT3 activation promotes the expression of some antiapoptotic genes (e.g., Bcl-xL) and antioxidant proteins (e.g., superoxide dismutase), which protect the heart from I/R injury (Harhous et al., 2019). Compared with wild-type mice, cardiomyocytes-specific knockout of STAT3 induces much more serious injury in the heart following I/R, namely, increased apoptosis of cardiomyocytes and mortality (Hilfiker-Kleiner et al., 2004). The cardioprotective role of STAT3 was further confirmed by analyzing transgenic mice overexpressing active STAT3. Mice overexpressing STAT3 are more resistant to I/R-induced damage than wild-type mice and exhibited smaller infarct areas (Oshima et al., 2005).

#### 3.3.3 p53

As a proapoptotic transcription factor, p53 was first considered a tumor suppressor that also regulates the apoptosis process in cardiomyocytes. In rats with cardiac I/R injury, p53 accumulates and is activated by acetylation, which mediates the transcription of Bax, a cell death effector, to initiate the apoptosis program (Gogna et al., 2013). In addition to the upregulation of Bax, the downstream targets of Bax are also upregulated in a rat I/R model, including cytochrome C, apoptosis-inducing factor, cleaved caspase-3, caspase-9, and apoptotic peptidase activating factor 1, indicating the critical function of p53 in regulating cardiomyocyte apoptosis (Men et al., 2021). In human, the upregulated p53 protein expression is associated with heart failure (Song et al., 1999). Therefore, upregulated p53 mediates decompensated pathological cardiac hypertrophy through p53-dependent apoptosis, while downregulated p53 rescues cardiac hypertrophy (Men et al., 2021). Raut et al. (2016) found that decreased p53 expression by miR-30c or miR-181a overexpressing attenuated diabetes-induced cardiac hypertrophy, indicated by decreased ANP expression and cardiomyocytes size. Moreover, some researchers recently reported that p53 modulates the function of some noncoding RNAs to regulate the execution of apoptosis. For example, using MI mice model, Li X et al. (2019) found that long noncoding RNA Meg3, an apoptosis inducer controlled by p53, binds to FUS, an apoptosis inducer, to promote apoptosis through the upregulation of the proapoptotic protein caspase-9 and downregulation of the antiapoptotic protein Bcl-2. P53 has also been shown to participate in the endoplasmic reticulum (ER) stress-induced apoptosis pathway in cardiomyocytes. Cardiomyocytes-specific knockout of p53 decreases thapsigargin-induced ER stress and attenuates cardiomyocyte apoptosis (Chen et al., 2019). However, these inhibitory effects were incomplete, indicating that the other pathway may also participate in ER stress-induced mitochondrial damage.

#### 3.3.4 Forkhead Box Transcription Factor O Transcription Factors

The forkhead box transcription factor O (FoxO) family regulates diverse cellular signaling pathways in specific cells. Currently, four members of the FoxO family (FoxO1, FoxO3, FoxO4, and FoxO6) have been identified in mammalian cells. Emerging evidence has proven that the FoxO family controls the activation of apoptosis-related pathways by regulating the transcription of several genes, including Bnip3 and Bax (Sengupta et al., 2011). Using an oxidative stress NRVMs model, Sengupta et al. (2011) found that ROS-induced cardinal injury promotes the nuclear localization of both FoxO1 and FoxO3 (Sengupta et al., 2011). The activation of FoxO1 and FoxO3 subsequently initiates superoxide dismutase and CBP/P300-interacting trans-activator expression, which serve as antioxidant and antiapoptotic proteins, respectively, to protect cardiomyocytes from damage and apoptosis. Moreover, the apoptosis repressor with caspase recruitment domain, a potent anti-apoptosis protein, is a direct transcriptional target of FoxO3a (Lu et al., 2013). Upregulated FoxO3a suppresses the release of Ca^2+^ from the sarcoplasmic reticulum induced in response to oxidative stress to inhibit Ca^2+^-mediated apoptosis by increasing the level of apoptosis repressor with caspase recruitment domain in cardiomyocytes (Lu et al., 2013).

#### 3.3.5 GATA4

The critical role of the transcription factor GATA4 in cardiomyocyte apoptosis has been extensively investigated. Upregulated GATA4 is negatively correlated with cardiomyocyte apoptosis. Doxorubicin upregulates miR-208a to inhibit the expression of GATA4 and promote cardiomyocytes apoptosis in mice, while silencing of miR-208a reverses the increase in cardiomyocyte apoptosis by increasing Bcl-2 expression *via* GATA4 in mice (Tony et al., 2015). According to a subsequent study, GATA4 coordinates with cardiac ankyrin repeat protein to directly bind the GATA binding site located in the Bcl-2 promoter and upregulate Bcl-2 expression to counter doxorubicin-induced cardiomyocyte apoptosis (Zhang et al., 2016).

### 3.4 Regulation of Autophagy

Proteins and organelles are continuously synthesized throughout the cell lifespan. The proper degradation and turnover of proteins and organelles play pivotal roles in homeostasis and survival of cells and life. Autophagy is an evolutionarily conserved subcellular pathway for lysosome-mediated turnover of proteins and organelles that was first discovered in 1963. Basal autophagy is crucial for cardiomyocytes to maintain hearts homeostasis and normal function, permitting the clearance and recycling of obsolete and dysfunctional elements (Sciarretta et al., 2018). In particular, the cells of terminally differentiated organs are unable to remove their waste without autophagy because they do not replicate (Sciarretta et al., 2018). This function of autophagy was highlighted using cardiomyocytes-specific knockout of autophagy-related proteins. Cardiomyocytes-specific inactivation of autophagy-related protein (ATG) 5, a crucial protein involved in autophagy, causes progressive heart failure through the accumulation of ubiquitylated proteins, mitochondrial dysfunction, and disorganized sarcomeres (Sciarretta et al., 2018). However, excess autophagy leads to cell death. The dual roles of autophagy suggest that the beneficial or detrimental function of autophagy in the heart depends on the type of stimulation and timing of the measurement. Basically, a suitable level of autophagy benefits the heart.

#### 3.4.1 Transcription Factor EB

Transcription factor EB (TFEB) plays a crucial role in regulating autophagy and lysosome biogenesis and is characterized by a basic helix-loop-helix-leucine zipper motif. Dephosphorylated TFEB translocates to the nucleus and binds to specific DNA sequences to regulate the expression of autophagy- and lysosome-related genes, such as ATG9, vacuolar protein sorting-associated protein 11, and lysosomal-associated membrane protein 1 (Settembre and Ballabio, 2011). TFEB nuclear translocation decreased in DCM patient, indicating the protective function of TFEB in heart (Caragnano et al., 2019). In young mice, increased TFEB translocation ameliorates lipopolysaccharide-induced oxidative damage by activating the autophagy cascade in cardiomyocytes, while TFEB does not translocate and initiate autophagy-related gene expression in aged mice treated with lipopolysaccharide (Li et al., 2016). Similarly, glucolipotoxicity caused by diabetes and obesity in mice reduces TFEB expression in cardiomyocytes and leads to a disruption of lysosomal homeostasis, impaired cardiomyocyte proteostasis, and cardiac myopathy (Trivedi et al., 2016). TFEB overexpression protects the heart from cardiac proteotoxicity by increasing autophagy flux (Pan et al., 2017). After treatment with 3-methyladenine, a type III phosphatidylinositol 3-kinase inhibitor used to suppress autophagy, the protective effect of forced expression of TFEB vanished in NRVMs, indicating the protective effect of increased autophagy flux induced by the TFEB transgene (Pan et al., 2017). Recently, Trivedi et al. (2020) found that the protective function of TFEB in cardiomyocytes bypassed the autophagy process. The cardiomyocyte-specific deletion of TFEB aggravates nutrient overload-induced lipid droplet accumulation and caspase-3 activation, while TFEB overexpression in NRVMs exerts the opposite effect. In the presence of ATG7 loss-of-function, the protective effect of overexpressed TFEB persists, indicating that the effect of TFEB on reprogramming energy metabolism was more evident than its effect on regulating autophagy to protect cardiomyocytes (Trivedi et al., 2020). Targeting TFEB to maintain a suitable autophagy level in the diseased heart may be a novel idea for new drug development.

#### 3.4.2 Nuclear Factor κB

In addition to the apoptotic pathway, NF-κB also plays a pivotal role in autophagy. The well-known target of NF-κB in autophagy is Beclin-1. In rabbit I/R-injured hearts, Beclin-1 upregulation, which is induced by active p62, aggravates cardiac dysfunction, especially in the cardiac area at risk (Zeng et al., 2013). Overexpression of IκB, an inhibitor of the NF-κB signaling pathway, reduces p65 nuclear translocation and is associated with decreased Beclin-1 expression and a reduced LC3-II/LC3-I ratio in mice exposed to H_2_O_2_, suggesting that the reduced autophagy level protects the heart from H_2_O_2_-induced oxidative stress (Han et al., 2020). A similar outcome was confirmed in the diabetic mice. Increased autophagy activation damaged the hearts of diabetic mice, while a p65 siRNA significantly ameliorated this injury (Wang et al., 2017).

#### 3.4.3 Forkhead Box Transcription Factor O

Many studies have confirmed that FoxO induces the expression of multiple autophagy-related proteins, including ATG8, ATG12, ATG4B, phosphatidylinositol 3-kinase catalytic subunit type 3, GABA type A receptor associated protein like 1 and Beclin-1 (Ferdous et al., 2010). FoxO overexpression in NRVMs attenuates agonist-induced cardiac hypertrophy and increases autophagy levels. In energy-deprived cardiomyocytes, activated FoxO1 promotes autophagy-related gene expression and stimulates autophagy flux (Hariharan et al., 2010). Nonetheless, the beneficial or detrimental role of FoxO in cardiomyocytes is difficult to determine. In human, increasing FoxO3a expression was noted in heart from heart failure patients compared with control group (Galasso et al., 2010). In mice ischemic hearts, poly (ADP-ribose) polymerase-1 promotes FoxO3a activation and translocation (Wang et al., 2018). Subsequently, the activation of FoxO3a increases autophagy-related gene expression, which further impairs mitochondrial function, increases cell death, and aggravates pathological cardiac remodeling (Wang et al., 2018).

### 3.5 Regulation of Fibrosis

In addition to cardiomyocytes, fibroblasts are an important component of the heart and contribute to 10–30% of the total cardiac cell population. Fibroblasts are mainly responsible for providing basic structural support by ECM into the myocardium. Therefore, fibroblasts play a central role in tissue repair after injury. In damaged heart, cardiac fibroblasts express excessive cytokines and growth factor, leading to excessive proliferation of cardiac fibroblasts, myofibroblasts activation, and excessive secretion of ECM (Travers et al., 2016). Transcriptome changes are required for fibroblast transformation. Myofibroblasts express numerous contractility-related genes, including *Acta2* and *Tagln*, and secrete abundant ECM components, such as collagen I and III, fibronectin 1, tenascin-C, periostin, and matrix metalloproteinases (Travers et al., 2016). However, unlike other tissues, abundant ECM deposition does not stimulate cardiomyocytes to repair injury due to the terminal differentiation of cardiomyocytes. Additionally, the transformed myofibroblasts do not return to quiescent fibroblasts. Notably, after acute injury, the activated cardiac fibroblasts not only secret excessive ECM in the damaged site, but also produce large amount of ECM in remote healthy tissue, which reduces tissue compliance and progress into overt heart failure (Travers et al., 2016). Therefore, although early- and short-term cardiac fibrosis adapts to heart remodeling, persistent fibrosis is still detrimental to cardiac function.

#### 3.5.1 Small Mothers Against Decapentaplegic Transcription Factors

Small mothers against the decapentaplegic (SMAD) is a critical transcription factor family controlling multiple genes expression in myofibroblast, such as *Col1a*, *Acta2*, and *Tagln* (Lighthouse and Small, 2016). The SMAD family consists of three subtypes with different transcriptional functions: the activating SMADs (1, 2, 3, 5 and 8), the inhibitory SMADs (6 and 7), and the cofactor SMAD4. Additionally, among these activating SMADs, they are activated by different signals. Briefly, SMADs 1, 5 and 8 are generally activated by bone morphogenetic proteins while SMADs 2 and 3 are often referred to transforming growth factor-β (Hanna et al., 2021). In conical TGF-β pathway, activation of either BMP or TGF-β leads to SMADs activation and fibroblast activation through TβRI. Notably, some researchers reported that non-conical TGF-β pathway also played critical roles in activating SMADs and ECM synthesis through other signaling molecular but not TβRI, such as Rho-associated protein kinase (Burch et al., 2011; Kamato et al., 2013). Directly loss-of-function or gain-of-function studies also revealed the important regulating function of SMADs in fibrosis and ECM synthesis. In SMAD3-deficient mice, cardiac fibroblasts produce less collagen and included fewer activated fibroblasts characterized by *Acta2* positivity than wild-type mice (Dobaczewski et al., 2010). Furthermore, cardiac remodeling induced by myocardial infarction (MI) and diabetes mellitus are attenuated by the loss of SMAD3 in mice (Dobaczewski et al., 2010). Recently, Zhou et al. (2019) found that the Notch signaling pathway antagonizes SMAD3 activation and inhibits fibroblast transformation. Similar to SMAD3, cardiac fibroblast-specific loss of SMAD2 transiently ameliorates postinfarction-induced cardiac hypertrophy with preserved ventricular function (Huang et al., 2019). However, cardiac fibroblast-specific knockout of SMAD2 does not alter collagen expression (Huang et al., 2019). Additionally, SMAD3, but not SMAD2, regulates the formation of organized myofibroblast arrays and controls integrin and GTPase RhoA expression (Huang et al., 2019). Notably, inhibitory SMAD7 is downregulated in hearts of rats with MI (Wang et al., 2007). Several SMAD7-related noncoding RNAs have been identified in cardiac fibroblasts, such as miR-21, miR-216a, and lncRNA-Ang362, which promote cardiac fibrosis in various animal models by suppressing SMAD7 expression (Yuan et al., 2017; Tao et al., 2019; Chen et al., 2020).

#### 3.5.2 Myocardin-Related Transcription Factor

Recent studies have revealed the predominant function of myocardin-related transcription factor (MRTF) in cardiac fibrosis, which is encoding by megakaryoblastic leukemia genes. In the quiescent state, MRTF is broadly expressed and sequestered in the cytoplasm by binding G-actin. Stress stimuli, including hypoxia, inflammation, and ROS, induce the polymerization of F-actin and alterations in these connections, permitting the nuclear translocation of MRTF. Subsequently, the translocated MRTF forms a complex with SRF to bind CArG elements in the promoters of several genes related to fibrosis. Blockade of MRTF-A reduces scar and fibrosis formation after MI or angiotensin Ⅱ treatment in mice (Small et al., 2010; Francisco et al., 2020). Additionally, MRTF-A-null mice display decreased expression of genes involved in extracellular matrix production and smooth muscle cell differentiation, especially *Col1a2* expression (Small et al., 2010). Therefore, approaches targeting MRTF appear to be a good and new therapeutic method for treating cardiac injury. Indeed, many small-molecule MRTF inhibitors have been exploited and shown good performance in treating local fibrosis in various tissues, but not in the heart (Yu-Wai-Man et al., 2017). The strategy of treating cardiac fibrosis by targeting MRTF is worth exploring.

#### 3.5.3 Nuclear Factor of Activated T-Cells

Similar to cardiomyocytes, the calcineurin/NFAT signaling pathway is critical for fibroblasts. The dephosphorylation and translocation of NFAT triggers fibrotic gene expression in cardiac fibroblasts, including *Col3* and MRTF-A (Herum et al., 2013). In pressure-overloaded mice, upregulated calcineurin/NFAT contributes to cardiac fibrosis formation (Wang et al., 2016). Interestingly, regardless of the pharmacological inhibition of calcineurin by cyclosporin A or mutations in calcineurin, NFAT translocation is downregulated and cardiac fibrosis is ameliorated in PE-treated primary rat cardiac fibroblasts (Wang et al., 2016). Currently, an increasing number of researchers have focused on signaling upstream of calcineurin/NFAT through a Ca^2+^ transporter called transient receptor potential (TRP). TRPA1 expression is required for TGF-β-induced fibroblast activation in a calcineurin/NFATc3-dependent manner (Li S et al., 2019). Similarly, in addition to TRPC3, TRPC6, TRPM6, TRPM7, TRPA1, TRPV3 and TRPV4 have been reported to directly participate in cardiac fibrosis progression by regulating the intercellular Ca^2+^ concentration (Inoue et al., 2019).

#### 3.5.4 SRF

In cardiac fibroblasts, function of SRF have been extensively studied. SRF activates cardiac fibroblasts *via* promoting several genes transcription, such as *Acta2* and *Col1* (Bretherton et al., 2020). As mentioned above, MRTF is a key factor for SRF transcriptional activation. Cleaved rho-associated coiled-coil protein kinase 1 isoform (N-terminal part) promotes SRF to initiate the targeted gene, TGF-β1, transcription by releasing MRTF from G-actin, which causes cardiac fibroblasts activation subsequently (Yang et al., 2012). Besides Rho/MRTF pathway, MAPK/Extracellular regulated protein kinases (ERK) 1/2 pathway also participates the activation of SRF. The ERK1/2-activated SRF promotes cellular inhibitor of apoptosis 2 expression in cardiac fibroblast. Cell cycle of cardiac fibroblasts is also regulated by SRF activation. The activated SRF up-regulated S-phase kinase-associated protein 2 expression, which facilitates post-translational degradation of p27, a cyclin-dependent kinase inhibitor arresting cell cycle, to promote G1-S transition and cardiac fibroblast proliferation (Titus et al., 2020). It is still unclear how ERK1/2 influence SRF activation in cardiac fibroblasts. However, it has been reported that this activation effect is mediated by ternary complex factors in embryo fibroblast, which competitively inhibits MRTF binding with SRF (Gualdrini et al., 2016). Similar mechanism may happen in cardiac fibroblasts, further explorations are needed.

#### 3.5.5 Transcription Factor 21

Cardiac fibroblasts are thought to be predominantly derived from the epicardial cells (Deb and Ubil, 2014). Through a process called epithelial-to-mesenchymal transition (EMT), some epicardial cells migrates into the myocardium and differentiate into coronary vascular smooth muscle cells or cardiac fibroblasts. During EMT process, transcription factor 21 (TCF21) express in epicardial cells. Genetic lineage tracing study showed that TCF21 specifically expresses in cardiac fibroblasts lineage, which is essential for the cell fate determination of cardiac fibroblasts in development and remains active in adult mice (Ma et al., 2017). Therefore, TCF21 is often referred as a biomarker for tracing cardiac fibroblasts in developmental and disease study. Additionally, the inducible TCF21^mERCremER^ transgenic mice, which specifically express Cre recombinase in cardiac fibroblasts after treatment with tamoxifen, represents as a powerful mice line for studying cardiac fibroblasts (Travers et al., 2016). After acute injury, TCF21-expressing epicardial cells undergo EMT to generate cardiac fibroblasts, which express collagen and contribute to pro-fibrotic repair response, indicating TCF21 expression play critical role in the cardiac fibrosis (Acharya et al., 2012; Deb and Ubil, 2014).

## 4 Conclusion and Perspective

Many transcription factors, their target genes, protein interactions and biological functions have been recognized in the heart. Classically, transcription factors govern the expression of several critical proteins to influence related cellular processes. In addition to the canonical function of transcription factors in controlling functional protein expression, emerging evidence proves that they also play critical roles in epigenetics in the heart. Therefore, the recognition of transcription factors would help us to explore the cardiac disease process. Indeed, with the help of next-generation sequencing technology, patients’ genetic patterns have been characterized gradually. Mutations in multiple genes have been found to be directly associated with diseases, including cardiovascular diseases. Transcription factors, as directly upstream molecules regulating gene expression, might be an effective target for developing new cardiovascular drugs. In fact, some molecules from natural products have been proven to target transcription factors and treat heart diseases. However, few drugs ultimately enter the clinic and save patients’ lives. In this article, we summarized some extensively studied transcription factors involved in various cellular pathways in the heart, such as fetal reprogramming, energy metabolism, apoptosis, autophagy and fibrosis, and promise to provide insights for the further exploration of the mechanism and drug research for heart diseases in the future.

## Glossary

ANP: atrial natriuretic peptide
ARC: apoptosis repressor with caspase recruitment domain
ATG: autophagy-related protein
BNP: brain natriuretic peptide
Csx: cardiac-specific homeobox
DCM: dilated cardiomyopathy
ECM: extracellular matrix
EMT: epithelial-to-mesenchymal transition
ER: endoplasmic reticulum
ERK: extracellular regulated protein kinases
FAO: fatty acid oxidation
FoxO: forkhead box transcription factor O
HDAC: histone deacetylase
IκB: inhibitor of nuclear factor-κB
IKK: inhibitor of nuclear factor-κB kinase
I/R: ischemic/reperfusion
MEF2: myocyte enhancer factor 2
β-MHC: β-isoform of cardiac myosin heavy chain
MAPK: mitogen-activated protein kinase
MRTF: myocardin-related transcription factor
MI: myocardial infarction
NRVM: neonatal rats ventricular myocytes
NFAT: nuclear factor of activated T-cells
NF-κB: nuclear factor-κB
PPAR: peroxisome proliferator activator receptor
ROS: reactive oxygen species
SMAD: small mothers against decapentaplegic
SRF: serum response factor
STAT: signal transducer and activator of transcription
TAC: transverse aortic constriction
TCF21: transcription factor 21
TGF-β: transforming growth factor-β
TFEB: transcription factor EB
TRP: transient receptor potential
